# Genetic structure of Ethiopian finger millet landraces and genome-wide association mapping for agronomic and nutritional traits

**DOI:** 10.1007/s00122-025-04892-1

**Published:** 2025-05-08

**Authors:** Adane Gebreyohannes, Hussein Shimelis, Davis M. Gimode, Prasad Grandham, Vinod Kumar Valluri, Habte Nida, Susan M. Moenga, Chris O. Ojiewo, Benjamin Kilian, Damaris A. Odeny

**Affiliations:** 1 African Centre for Crop Improvement, School of Agricultural, Earth and Environmental Sciences, University of KwaZulu-Natal, Scottsville, 3209 South Africa; 2 Ethiopian Institute of Agricultural Research, Melkassa Agricultural Research Center, P.O. Box 436, Adama, Ethiopia; 3 International Crops Research Institute for the Semi-Arid Tropics, P.O Box 39063, Nairobi, 00623 Kenya; 4 Center of Excellence in Genomics and Systems Biology, ICRISAT – Patancheru, Hyderabad, 502324 India; 5 Department of Agronomy, Purdue University, West Lafayette, IN 47907 USA; 6 International Maize and Wheat Improvement Center (CIMMYT), ICRAF House United Nations Avenue, Gigiri, Village Market, P.O. Box 1041, Nairobi, 00621 Kenya; 7 Global Crop Diversity Trust, Bonn, Germany; 8 CGIAR Standing Panel on Impact Assessment, Alliance of Bioversity International and CIAT, Cali, Colombia; 9 School of Plant, Environmental and Soil Sciences, Louisiana State University Agricultural Center, Baton Rouge, LA USA

## Abstract

Finger millet (*Eleusine coracana* subsp. *coracana*) (2n = 4x = 36) remains one of the most important millets in East Africa (EA), where it was most likely domesticated along the highlands of Ethiopia and Uganda. The goal of the current study was to understand the population structure of the Ethiopian finger millet landraces and identify quantitative trait nucleotides (QTNs) and haplotypes associated with agronomic and nutritional traits. In a field evaluation across three environments, 448 genotypes were assessed for days to flowering (DTF), days to maturity (DTM), thousand seed weight (TSW), grain yield (GY), stay-green score (STG), and drought score (DrtSc). The harvested grain was analyzed for Fe and Zn contents. A subset of 391 genotypes was skim-sequenced, generating 24,112 high-quality SNPs that were employed for population structure, association mapping, and haplotype analysis. Seventy marker-trait associations were detected including 15 major QTNs with more than 30% phenotypic variance explained (PVE) for all traits except STG and GY. Pleiotropic major QTNs were identified for DTM/DTF and Fe/Zn on chromosomes 9B and 2B, respectively. Haplotype analysis of major QTNs identified 54 significant haplotype blocks and 2 additional haplotypes for a *multidrug ABC transporter gene family like* protein on chromosome 4A that was associated with PTH. Favorable haplotypes from pleiotropic DTM/DTF and Fe/Zn QTNs were present in 13 and 12 genotypes respectively, majority from Tigray region. Two genotypes from Tigray and one from Amhara harbored favorable haplotypes for DTM/DTF and Fe/Zn. These findings provide invaluable insights for targeted breeding to enhance finger millet resilience, nutritional profile, and yield.

**Supplementary Information:**

The online version contains supplementary material available at 10.1007/s00122-025-04892-1.

## Introduction

Finger millet {2n = 4x = 36; *Eleusine coracana* (L.) Gaertn} is an important cereal crop cultivated on ~ 2.1 m ha with an annual production of ~ 3.7 m tons globally (Indiastat 2020; FAOSTAT 2021). It represents 20% of the global millet area and 26% of the total global millet production, respectively (Gebreyohannes et al. 2024). It is the most important millet in East Africa, where it is believed to have been domesticated along the sub-humid highlands (Hilu et al. 1979; Tsehaye et al. 2006; Tesfaye and Mengistu 2017). Its adaptability to extreme weather conditions, extended storage quality, rich nutritional profile, high seed multiplication rate, and diverse health benefits make finger millet a desirable climate resilient crop for food and nutrition security (Ceasar et al. 2018; Kudapa et al. 2023; Gebreyohannes et al. 2024).

Finger millet exhibits outstanding adaptability due to its C4 photosynthetic pathway. This metabolic advantage, as documented by Yogeesh et al. (2016), Hittalmani et al. (2017), Ceasar et al. (2018), and Kudapa et al. (2023), allows finger millet to thrive in diverse and often challenging climates characterized by high temperatures, intense heat, and limited moisture availability. Beyond its climatic resilience, finger millet demonstrates additional stress tolerance. Studies by Hatti et al. (2018) report its ability to perform well in depleted soils, while Krishnamurthy et al. (2014) highlight its salinity tolerance. This inherent adaptability is further emphasized by Dida and Devos (2006), who describe finger millet's success in various agro-ecological zones, performing well under low rainfall in drylands and on a wide range of tropical soils.

Finger millet boasts an exceptional nutritional profile, which is particularly rich in a range of nutrients such as calcium (50–589 mg/100 g), potassium (430–669 mg/100 g), iron (3–53 mg/100 g), phosphorus (130–250 mg/100 g), sulfur (106–149 mg/100 g), sodium (6–42 mg/100 g), and magnesium (78–201 mg/100 g) (Backiyalakshmi et al. 2023; Teklu et al. 2024). This impressive array of nutrients, along with rich sources for essential amino acids, dietary fiber (18%), and phenolic compounds (0.3–3%), has earned finger millet the title as a"superfood"(Devi et al. 2014; Krishnan 2024). The grain is gluten-free and has extended storage quality due to the polyphenol content (Xiang et al. 2019; Odeny et al. 2020; Gebreyohannes et al. 2021), thus an important cereal for famine reserves. After grain harvest, the finger millet stover is an important source of fodder for livestock, enhancing feed security in areas that rely on crop and livestock farming. Micronutrient deficiencies, often referred to as the hidden hunger, affect over 2 billion people worldwide, primarily in low-income developing countries (Gupta et al. 2017; Sheoran et al. 2022; Teklu et al. 2023a). Micronutrient deficiencies are particularly severe in sub-Saharan African countries, where many populations lack access to a diverse diet. A recent study by Gebremedhin et al. (2021) reported that in Ethiopia, 37.3% of children aged 6–23 months had not received any recommended micronutrient sources. Therefore, finger millet is a healthy alternative for managing nutrition deficiencies, especially when the affected families adopt and cultivate high micronutrient-containing varieties.

While finger millet boasts climate resilience and impressive nutritional value, its production is severely hampered by several biotic and abiotic challenges. Yield losses exceeding 50% have been reported due to drought stress, impacting both grain quality and key yield components (Maqsood and Ali 2007). Furthermore, the genetic control of the important agronomic traits is not fully understood. Variability for the desirable nutritional traits and how they are affected by different soils and environments are equally not well studied. There have been hypotheses suggesting that the micronutrient content may be negatively correlated with yield (Upadhyaya et al. 2010; Backiyalakshmi et al. 2023) but there are no convincing and reproducible experiments reporting the same. The current study employed 448 diverse finger millet genotypes to enhance the understanding of genetic factors controlling agronomic and nutritional traits. The genotypes were evaluated in three different environments for both agronomic and nutritional traits. High throughput genotypic data was generated through skim-sequencing of 391 genotypes and a subsequent comprehensive genome-wide association study (GWAS) of all traits undertaken using different mixed models. The GWAS identified significant high-effect quantitative trait nucleotides (QTNs) for agronomic, drought, and tolerance related traits and Fe and Zn contents.

## Materials and methods

### Description of the experimental sites

Three different sites were used in the study; Arsinegelle (E1), Maitsebri (E2), and Meiso (E3) in Ethiopia (Supplementary Fig. S1). E1 is characterized by loam soils, slightly acidic pH, higher total organic carbon and nitrogen content, moderate clay content, and relatively high annual rainfall. E2 experiences moderate annual rainfall. E3 is characterized by clay loam soil, neutral pH, lower total organic carbon and nitrogen content, highest clay content, and lowest annual rainfall (Supplementary Table S1). The study areas varied in altitudes, which spanned from 1323 to 1933 m above sea level. Before planting, soil samples were taken across the three sites from various depths ranging from 0 to 60 cm. The soil samples were thoroughly mixed and transported to Melkassa Agricultural Research Center (MARC, Adama, Ethiopia) for physicochemical analysis employing the Estefan et al. (2013) method. Nutritional composition analysis was also carried out at MARC, at the food and nutrition laboratory immediately following harvest.

### Plant material

A comprehensive collection of 448 finger millet accessions, including 425 diverse landraces and 23 improved varieties was assembled for the present study (Supplementary Table S2). The germplasm was sourced from various regions and research centers across Ethiopia, representing the country's rich diversity in finger millet (Supplementary Fig. S1). Tigray contributed the highest number of germplasm collection (168 entries), followed closely by Amhara (145 entries) and Oromia (86 entries), the Southern Nation Nationalities People Region (SNNPR; 17 entries) and Benishangul and Gumuz (9 entries) (Supplementary Table S2). The study additionally included 23 released crop varieties developed by the Ethiopian regional and national research institutes, encompassing research centers, namely, Bako, Melkassa, Pawe, Adet, Jinka, Shire-Maitsebri, and Axum (Supplementary Table S2).

## Experimental design, planting, and managing the field experiments

A row-column design with two replicates was employed at each location with each replicate consisting of 64 rows and 7 columns. Each plot consisted of three meters of a single row with a row spacing of 0.4 m and a plant-to-plant spacing of 10 cm. To minimize lodging risk, only Di-ammonium phosphate (DAP) fertilizer was applied during sowing at the rate of 100 kg/ha. No additional nitrogen fertilizer (urea), herbicide, and insecticide were applied during the subsequent growth stages. Weeding was performed manually.

### Phenotyping for agro-morphological traits

Agro-morphological and drought related data were collected following the procedures already described in IBPGR (1985) with minor modifications. Plant height (PTH; cm) was measured from ground level to the tip of the inflorescence (ear) at the dough stage on five randomly selected plants within each plot. Days to 50% flowering (DTF) was recorded as the number of days from sowing to when half of the main tillers in a plot had emerged ears. Similarly, days to maturity (DTM) were recorded as the number of days from sowing to the day when the main tillers in a plot had fully mature ears. Thousand seed weight (TSW) was determined by drying 1000 randomly chosen seeds to a standard moisture content of 12.5% and recording their weight in grams (g). Grain yield (GY; t ha^−1^) was measured as the total grain weight of a plot (1.2 m^2^) harvested at 12.5% moisture content and then converted to t ha^−1^. Stay-green score (STG) was rated using a visual score on a scale of 1 to 5 per plot basis representing plant canopy greenness at maturity, with 1 indicating fully stay green (drought tolerant) (Supplementary Fig. S2), and 5 indicating completely senesced (drought sensitive). Finally, drought score (DrtSc) was assessed using a visual score on a scale of 1–5 per plot basis, representing overall drought tolerance, where 1 refers to highly tolerant, and 5 indicating highly sensitive. It is essential to note that drought tolerance is a complex trait encompassing multiple physiological and morphological attributes, including stay green, earliness, and late maturity with associated stay-green characteristics.

### Phenotyping for nutrient content

Nutritional composition analysis of the crop was conducted at MARC, at the food and nutrition laboratory immediately after harvest. A representative grain sample of 150–200 g per accession was carefully collected from each replicated plot at each testing site during the 2019/2020 harvesting period. The seeds were cautiously inspected to ensure they were clean and free from any inert matter, dust, or grains from other crops. To prevent moisture loss and contamination, the clean seeds were stored in airtight containers. Prior to near infra-red (NIR) analysis, a standardization step was performed using a reference plate made of polystyrene, a material with a known NIR spectrum. This step calibrated the instrument to match the NIR spectrum of the reference plate, ensuring accurate NIR spectrum measurements of the samples. For non-destructive NIR analysis, a representative amount of the seed sample (150–200 g) was added into the sample hopper of the NIR analyzer (Perten Instruments AB, Sweden). The NIR analysis process was then initiated, and the Perten IM 9500 measured the NIR spectrum of the sample. This measurement process was repeated twice, and the average of the two readings was used for each sample to enhance accuracy. The machine recorded data on iron (Fe) (mg/100 g), and zinc (Zn) (mg/100 g) contents.

### Leaf tissue sampling, DNA extraction, library preparation and sequencing

To guarantee seed purity and uniformity, three rounds of single-seed descent were conducted for each genotype before phenotyping. Subsequently, 3–4 seeds were sown in six ‘72 cavity seedling trays’ at MARC, Ethiopia. Thinning was done to ensure only one healthy and vigorous seedling was retained per cavity. Leaf tissue samples were collected 3–4 weeks after planting and placed in individual wells of a 96-well plate for efficient sample handling and storage. The collected samples were deep-frozen at − 80 °C for 24 h, before being subjected to freeze-drying (lyophilization) using the Martin Christ Alpha 1–2 LDplus (Donau lab, Ukraine) for 24 h. The lyophilized leaf samples were carefully packaged and shipped to the International Crops Research Institute for the Semi-Arid Tropics (ICRISAT) genomics laboratory unit in Nairobi, Kenya for DNA extraction. DNA was extracted using the Isolate II plant DNA extraction kits (Bioline Pty Ltd, Nottingham, UK) according to manufacturer’s instructions. The purity and quantity of the extracted DNA were determined using gel electrophoresis and a Qubit 2.0 Fluorometer (Life Technologies, Carlsbad, CA) respectively with final dilution to 50 ng/μl. DNA samples were sent to Psomagen (USA) for library preparation and next-generation sequencing. Libraries were constructed using the RIPTIDE kit (Twist Bioscience, San Francisco, CA, USA) according to the manufacturer’s protocol. The libraries were subjected to paired-end sequencing on the Illumina Novaseq6000.

### Quality control and SNP calling

Raw sequencing reads were trimmed to remove adapter sequences and poor quality reads using Trimmomatic (version 0.39) as per Bolger et al. (2014). The high-quality reads were aligned to the chromosome scale finger millet reference genome (Devos et al. 2023) and SNPs called using GATK (version 4.2.5.0) (McKenna et al. 2010). Haplosweep, a tool specifically designed for filtering high-quality SNPs from polyploid crops was used to eliminate homeologous SNPs (Clevenger et al. 2018). Additional filtering was done to retain SNPs with a minor allele frequency (MAF) > 5%, < 20% missing data, and heterozygosity of less than 25%. Only SNPs that mapped to finger millet’s 18 chromosomes were retained for downstream analysis.

### Population structure and linkage disequilibrium

Filtered SNPs were used to undertake principal component analysis (PCA) in the SNP & Variation Suite (SVS version 8.9.0). To establish the differences and likely effects of each of the finger millet sub-genomes, whole-genome SNPs were first used for a separate PCA analysis. Subsequently, additional analyses were conducted using SNPs generated from sub-genomes A and B. Ten principal components (PCs) and the additive model were used to generate Eigen values. The first three principal components of the variation were plotted and visualized in R software using the scatterplot3 d 0.3–41 package (Ligges and Machler 2003). The Discriminant Analysis of Principal Components (DAPC) was done using the adegenet v. 2.1.10 package in R software by retaining the first three principal components (Jombart 2010). Linkage disequilibrium (LD) decay was also done for whole genome, sub-genomes A and B SNPs independently. LD decay was estimated by using LD analysis from Tassel v5 (Bradbury et al. 2007) program with a sliding window of 10 kb. The resulting LD *R*^2^ values were plotted using R script. The *r*^2^ threshold was set to 0.2.

### Phenotypic data analysis

Analysis of variance (ANOVA), means, and variances for each quantitative trait was done using R (R core team 2021). Best linear unbiased predictions (BLUPs) and thereafter variance components within environments were estimated in the lme4 package (Bates et al. 2015) in R (R core team 2021) by manipulating the REstricted Maximum Likelihood (REML) method using the model:

$$Y_{ijk} = \mu + r/\alpha i + r/\beta_{j } + \varepsilon_{ijk}$$

where *Y*_*ijk*_ is the *k*th observation for the *i*th row and *j*th column; *µ* is the overall mean; $r/\alpha i$ and r/βj are the effect of *i*th and *j*th rows and columns nested in replicates, respectively.

ε_ijk_: Random error term.

BLUP variance components estimated within environments were appropriated to calculate broad-sense heritability (*H*^2^) for respective traits per location using the formula:

*H*^*2*^ scores were classified according to Robinson et al. (1949) as follows: 0–30% = low; 30–60% = moderate; > 60% = high. BLUPs were further used to generate frequency distribution curves and input as trait in GWAS. Pearson's correlation coefficients between all possible trait pairs were analyzed using the Proc corr function in SAS (SAS 2008).

### Marker-trait associations

Marker-trait associations (MTAs) were calculated by combining the filtered SNP dataset of the genotypes with the corresponding BLUPs in R software using the Genome Association and Prediction Integrated Tool (GAPIT) version 2 package (Tang et al. 2016). A combination of statistical models was employed, including BLINK, CMLM, and MLM. All the models used the first three principal components (PCs) as the fixed effect to correct for population structure. Manhattan and quantile–quantile (Q–Q) plots were generated using the R package qqman (Turner 2018) to visualize the results. Manhattan plots provide a scatter plot representation of the GWAS results, displaying the distribution of SNPs across the chromosome, while Q–Q plots assess the effectiveness of the GWAS model in accounting for population structure and familial relationships. A stringent Bonferroni-corrected *P* values (0.05/no. of markers) were used as the threshold for retaining markers with significant associations. Putative candidate genes within LD distance of the significant SNPs were identified based on the KNE796-1 reference genome available in Phytozome (https://phytozomenext.jgi.doe.gov/info/Ecoracana_v1).

### Haplotype analysis

The most rapid LD decay distance obtained after LD analysis was used to build haplotype blocks around all major quantitative trait nucleotides (QTNs). All markers that were within the LD decay distance of 1.2 Mb (600 Kb up- and downstream) of the major QTN were considered for the analysis based on the minimum LD distance calculated (Supplementary Fig. S3). Genes that were colocalized with a minimum of two SNPs around the major QTN haplotype blocks were analyzed for gene-specific haplotypes. Phenotypic data was categorized based on the identified haplotypes and used to test for haplo-pheno association in individual and across environments. One way ANOVA with Duncan's test as a post hoc test was used to identify significant associations and measure specific differences between pairs of means in R using package DescTools (Andri et al. 2021). Only haplotypes that were present in at least five genotypes were considered for the statistical analysis.

## Results

### Phenotypic variation

**Fig. 1:**
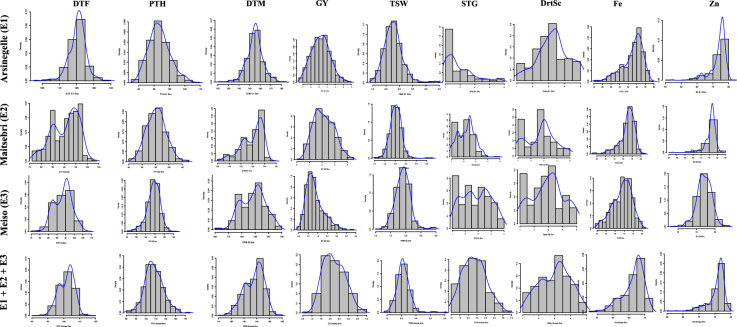
Frequency distribution graphs for each of the traits across all environments. The graphs were drawn using BLUPs

Highly significant differences were observed across locations (Loc), genotypes (Gen), and their interactions (Gen x Loc) for all traits under study at *P* < 0.001 (Table 1). DTF, DTM, GY, and DrtSc demonstrated high heritability across all environments (E1, E2, E3), with values ranging from 0.7 to 0.9. PTH and Fe content also displayed consistent moderate to high broad sense heritability across all environments (0.5–0.6). Mean DTF demonstrated marked environmental dependence, with E1 (120.8 days) and E2 (90.4 days) reporting contrasting extremes. The highest range and mean (0.2–6.6 tons/ha; 3.2 tons/ha) for GY was reported in E1 and the lowest (0.5–3.3 tons/ha; 1.2 tons/ha) from E3 (Table 1). Mean Fe and Zn reported similar and increasing patterns of variations from E3 to E1 (Table 1). Strong negative correlations were reported between phenological traits (DTF, DTM, STG, DrtSc) and yield components (TSW, GY) across all environments (Supplementary Table S3). Fe and Zn displayed consistent and location-independent positive association with correlation coefficients ranging from 0.76 to 0.81 (Supplementary Table S3). However, there was no correlation between Fe and Zn with all the agronomic traits, except for a once-off weak positive correlation (0.09) between Fe and GY at E3 (Supplementary Table S3). Frequency distribution histograms for all traits from each environment and across all environments are presented in Fig. 1.

**Table 1:** Descriptive statistics for all traits measured across the three environments

Location		DTF (days)	PTH (cm)	DTM (days)	GY (tons/ha)	TSW (g)	STG (1–5)	DrtSc (1–5)	Fe (mg/100 g)	Zn (mg/100 g)
Arsinegelle (E1)	Range	96.5–140.0	55.0–132.5	122.5–183.0	0.2–6.6	1.5–3.9	1–5	1–5	1.0–7.7	4.5–8.1
	Mean	120.8	86.6	155.1	3.2	2.3	1.9	3.3	5.5	7.4
	SE	1.9	7.0	2.9	0.2	0.1	0.4	0.4	0.7	0.2
	H^2^	0.8	0.6	0.8	0.9	0.8	0.8	0.8	0.5	0.7
Maitsebri (E2)	Range	76.0–103.5	62.3–146.5	103.5–145.5	0.3–4.5	1.0–3.7	1.0–4.7	1.0–5.0	2.6–8.2	5.2–8.3
	Mean	90.4	101.3	129.7	2.1	2.0	2.2	2.8	6.5	7.6
	SE	1.6	7.7	2.5	0.2	0.2	0.4	0.3	0.4	0.2
	H^2^	0.9	0.6	0.9	0.9	0.6	0.6	0.9	0.6	0.6
Meiso (E3)	Range	78.5–107.5	80.0–182.5	101.0–149.5	0.5–3.3	1.2–3.0	1.0–5.0	1.0–5.0	5.4–8.1	6.9–8.3
	Mean	93.5	136.4	128.6	1.3	1.9	2.9	3	6.9	7.7
	SE	93.5	136.4	128.6	1.3	1.9	2.9	3	6.9	7.7
	H^2^	0.8	0.6	0.8	0.7	0.7	0.8	0.8	0.6	0.5
Source of variation	Location	***	***	***	***	***	***	***	***	***
	Genotypes	***	***	***	***	***	***	***	***	***
	Gen x Loc	***	***	***	***	***	***	***	***	***

H^2^ = broad sense heritability; means were calculated based on best linear unbiased predictions (BLUPS); SE = standard error; Gen x Loc = genotype by location interaction, *** = significant difference at *P* < 0.001.

None of the top 10 best yielders in each of the environments was an improved variety except Mecha (229371) and Meba (GBK011119 A) at Meiso (Table 2). The top three best yielders in E1 surpassed the potential yield of 6 tons/ha and were from Tigray and Amhara (Table 2). Most of the top ten yielders in each of the locations were from Amhara and Tigray (Table 2). All the top 10 genotypes having the most abundant Fe and Zn were landraces, majority from Tigray (Table 3).

**Table 2:** A summary of the top ten best yielders in each of the environments and their origin

Arsinegelle (E1)			Maitsebri (E2)			Meiso (E3)		
Accession	Origin	Yield (tons/ha)	Accession	Origin	Yield (tons/ha)	Accession	Origin	Yield (tons/ha)
237468	Tigray	6.61	AxumARColl-22	Tigray	4.46	219819	Tigray	3.24
234170	Tigray	6.42	AxumARColl-21	Tigray	4.25	AxumARColl-37	Tigray	2.86
243617	Amhara	6.18	242115	Amhara	4.12	238326	Tigray	2.81
238325	Tigray	5.97	234186	Tigray	4.07	237449	Tigray	2.67
242122	Amhara	5.97	AxumARColl-28	Tigray	3.97	207897	Tigray	2.62
238297	Tigray	5.85	235835	Amhara	3.92	Mecha*	Amhara	2.62
234162	Tigray	5.73	243631	Amhara	3.89	234165	Tigray	2.56
238321	Tigray	5.69	AxumARColl-33	Tigray	3.88	234206	Tigray	2.53
237452	Tigray	5.64	235828	Amhara	3.80	Meba*	Oromia	2.48

*Improved varieties

**Table 3:** A summary of the top ten accessions with the highest Fe and Zn content in each of the environments

Environment	Accession	Origin	Fe (mg/100 g)	Accession	Origin	Zn (mg/100 g)
Arsinegelle (E1)	223145	Tigray	7.66	234159	Tigray	8.08
	242612	Tigray	7.41	234203	Tigray	7.99
	238319	Tigray	7.36	215982	Amhara	7.97
	235700	SNNP	7.35	243639	Amhara	7.97
	215805	Oromia	7.34	235700	SNNP	7.97
	238316	Tigray	7.33	238316	Tigray	7.97
	221697	Tigray	7.30	215805	Oromia	7.96
	238304	Tigray	7.24	221697	Tigray	7.95
	228306	Amhara	7.20	238319	Tigray	7.94
	229734	Amhara	7.16	223145	Tigray	7.94
Maitsebri (E2)	229735	Amhara	8.20	229735	Amhara	8.26
	215875	Amhara	8.10	215805	Oromia	8.16
	207963	Oromia	7.94	208730	Oromia	8.14
	234183	Tigray	7.80	238333	Tigray	8.13
	238323	Tigray	7.75	245085	Oromia	8.09
	238297	Tigray	7.68	238342	Amhara	8.09
	238317	Tigray	7.67	229728	Bennishangul & Gumuz	8.08
	234171	Tigray	7.63	219503	Amhara	8.05
	215805	Oromia	7.63	238313	Tigray	8.05
	223145	Tigray	7.61	238335	Tigray	8.02
Meiso (E3)	216048	Oromia	8.10	241769	SNNP	8.31
	234202	Tigray	7.94	242118	Amhara	8.25
	208730	Oromia	7.87	216032	Oromia	8.20
	238337	Tigray	7.84	100095	Oromia	8.18
	242122	Amhara	7.84	234154	Tigray	8.12
	211029	Tigray	7.84	215867	Amhara	8.11
	234207	Tigray	7.83	238313	Tigray	8.09
	234175	Tigray	7.81	242108	Amhara	8.09
	234171	Tigray	7.80	234193	Tigray	8.07
	243637	Amhara	7.80	216057	Oromia	8.04

### Linkage disequilibrium, genetic diversity, and population structure

Skim-sequencing generated 17,401,572 raw SNPs from 391 genotypes and retained 24,112 high-quality SNPs after filtering and LD pruning. Out of the 24,112 markers, 10,246 markers were from sub-genome A, while 13,866 were from sub-genome B. Assuming a genome size of 1.5 Gb, the overall density of the markers across the genome would be 16 SNPs/Mbp. LD varied depending on the SNP sets used. Whole-genome SNPs reported moderate LD decay at 2.1 Mb, with sub-genome B reporting the slowest decay point (3.2 Mb), and sub-genome A revealing the most rapid decay at 1.2 Mb (Supplementary Fig. 3). A detailed analysis of LD across markers of each chromosome is provided in Supplementary Table S4.

**Fig. 2:**
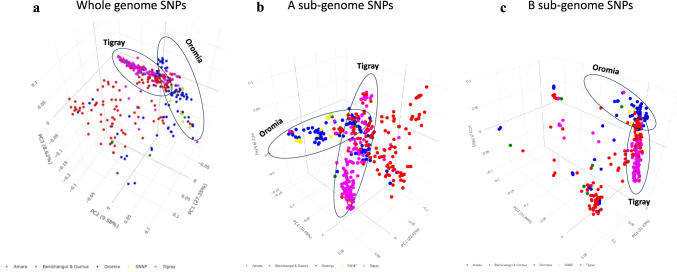
PCA using the retained markers across 391 genotypes. **a.** PCA done using whole-genome SNPs in which the first 3 PCs explain 45.6% genetic variation. **b.** PCA done using sub-genome A SNPs across the 391 genotypes where the first 3 PCs explain 43.3% genetic variation. **c.** PCA done using sub-genome B SNPs. The first 3 PCs explain 51.5 genetic variation across individuals

PCA using all markers and markers from each of the sub-genomes confirmed that the markers retained were informative and were able to differentiate the genotypes (Fig. 2). Sub-genome B markers were the most informative, explaining 51.5% genetic variation across the genotypes within the first 3 principal components (PCs), followed by whole-genome SNPs (45.6%) and lastly the sub-genome A SNPs (43.3%) (Fig. 2C). While most genotypes clustered as admixtures, there was relatively distinct clustering of genotypes from Oromia and Tigray regions (Fig. 2). Genotypes from SNNP region always clustered together with Oromia region genotypes, which is consistent with the neighboring locations of these two regions (Fig. 2 and Supplementary Fig. S1).

**Fig. 3:**
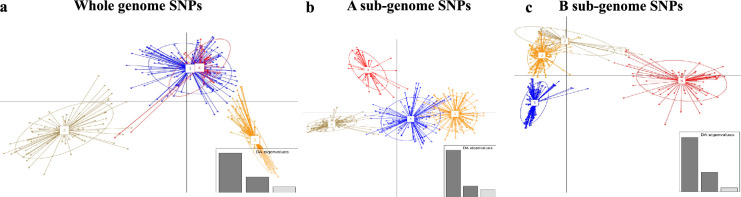
Discriminant analysis of principal components (DAPC) analysis using different marker categories. **a.** Whole-genome SNPs separated the genotypes into 4 sub-populations but sub-populations 1 and 4 were not distinct. **b** Sub-genome A SNP markers successfully separated the 4 sub-populations into distinct clusters. **c** Sub-genome B SNP markers distinguished clearly the sub-population 4 from the rest of the sub-populations. Membership in each of the clusters is described in Supplementary Table S5

To gain a deeper understanding of population structure, DAPC analysis was subsequently employed. The three categories of markers clustered the 391 genotypes into 4 sub-populations with different memberships (Fig. 3). Sub-populations generated by whole-genome SNPs were based on regions of origin, with clusters 2 and 3 being the most distinct and having genotypes predominantly from Amhara and Tigray regions, respectively (Fig. 3A), while cluster 1 was predominantly consisting of genotypes from Amhara (69 out of 145) (Supplementary Table S5). The most distinct sub-populations generated by the sub-genome A SNPs (Fig. 3B) was cluster 2 that consisted of majority genotypes from Tigray (100 genotypes), while genotypes from Amhara were split into 2 sub-populations (cluster 1 and 3) (Supplementary Table S5). Sub-genome B SNPs were the most informative in distinguishing genotypes from Tigray, which were grouped into cluster 1 (108 genotypes), but the same set of SNPs were not informative for genotypes from Amhara that were clustered in each of the four sub-populations (Fig. 3C; Supplementary Table S5). An overview of sub-population membership is presented in Supplementary Table S5.

### Genetic diversity of released varieties

**Fig. 4:**
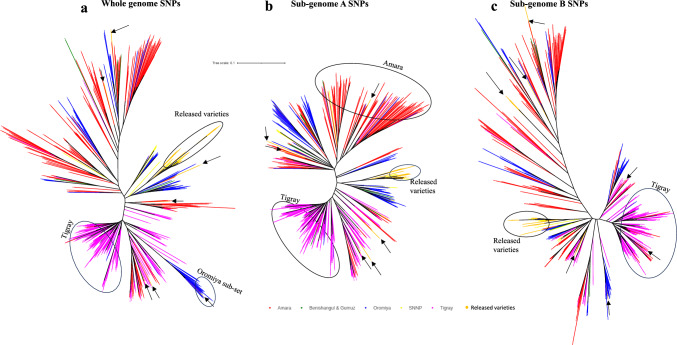
Dendrograms showing the clustering of released varieties in the background of all germplasm used in the current study. All released varieties clustering individually are shown with arrows. **a.** Clustering of released varieties using whole-genome SNPs. **b.** Clustering of released varieties using sub-genome A SNPs. **c.** Clustering of released varieties using sub-genome B SNPs

Although the different marker categories clustered most of the released varieties uniquely, they all revealed that the released varieties have been developed using a very narrow set of genotypic diversity, and almost always overlapped with genotypes from SNNP (Fig. 4). Whole genome SNPs showed a single cluster (16 out of the 22) of the released varieties that overlapped with genotypes from SNNP and was flanked by genotypes predominantly from Oromia (Fig. 4A). Sub-genome A SNPs followed a similar pattern and clustered most of the released varieties in between a sub-cluster of Oromia genotypes that also contained SNNP genotypes (Fig. 4B). Sub-genome B SNPs clustered the released varieties with an admixture set of genotypes from Oromia, SNNP, Amhara, and Benishangul & Gumuz (Fig. 4C). There were hardly any released varieties clustering under the predominant clusters from Tigray (pink; Fig. 4) and Amhara (red; Fig. 4) for both whole-genome and sub-genome A SNP markers. Although a cluster of Amhara genotypes was not as distinct using sub-genome B markers, there were not many overlaps between released varieties and Amhara genotypes (red; Fig. 4). The same scenario was observed for genotypes from Tigray, which might suggest that most of the collections from Tigray and Amhara are yet to be used in breeding programs for crop improvement.

### Marker-trait associations (MTA)

Seventy (70) MTAs were detected across the nine traits from at least one of the environments using the stringent Bonferroni threshold of *P* < 2.07366E-06 for whole-genome SNPs, *P* < 4.8E-06 for sub-genome A, and *P* < 3.6E-06 for sub-genome B (Table 4). Sixty-two of the 70 MTAs were detected using whole-genome SNPs while the remaining 8 MTAs were major (≥ 30% phenotypic variation explained) QTNs detected using sub-genome A and sub-genome B SNPs independently (Table 4). Out of the 70 MTAs, 8 markers were significant for more than one trait, resulting in a non-redundant set of 62 QTNs.

**Table 4:** Marker-trait associations detected using whole-genome and sub-genome SNPs across different models

Trait	SNP	*P* values			PVE (%)	Locations
		BLINK	CMLM	MLM		
*Using whole-genome SNPs*						
DTF	1 A_41691781	1.48E−06	–	–	11.8	E3
	6B_17245025	7.72E−07; 1.70E−07	–	–	2.0; 1.2	E2; E1 + E2 + E3
	7 A_42035020	3.38E−07	–	–	17.8	E1
	7B_29006305	1.31E−06	–	–	1.6	E1 + E2 + E3
	8 A_6485125	8.23E−10	–	–	6.7	E1
PTH	4B_50601501	6.51E−09; 1.30E−06	–	–	1.5; 4.8	E3, E1 + E2 + E3
	5B_27829128	1.29E−07	–	–	2.5	E3
	6 A_29685470	5.27E−07	–	–	16.1	E2
	6B_2712127	2.97E−08	–	–	2.1	E3
	7 A_35748697	9.979E−07	–	–	16.7	E1 + E2 + E3
	8B_59128846	9.16E−07	–	–	1.3	E3
	9 A_40793867	4.13E−07	–	–	1.0	E3
	9B_15553707	9.84E−09	–	–	9.0	E3
	9B_19836556	2.59E−09	–	–	8.4	E3
DTM	4 A_3946408	5.11E−08	–	–	7.3	E1
	**5B_65558510**	1.33E−06	–	–	40.6	E3
	6B_16874563	1.17E−06	–	–	10.3	E2
	6B_17245025	6.30E−08	–	–	1.4	E1 + E2 + E3
	7 A_42035020	2.72E−07	–	–	16.4	E1
	7B_29006305	8.81E−08	–	–	1.8	E1 + E2 + E3
GY	3 A_22037946	3.72E−09; 1.02E−06	–	–	7.2; 4.7	E1; E1 + E2 + E3
	4 A_40121282	1.25E−06	–	–	1.9	E1 + E2 + E3
	5B_55857122	2.61E−07	–	–	1.3	E1 + E2 + E3
	6 A_14427382	3.18E−11; 3.26E−11			10.6; 5.9	E1; E1 + E2 + E3
TSW	**1B_64659309**	–	3.77E−07	3.77E−07	56.1	E1 + E2 + E3
	1B_7786365	1.95E−06; 4.60E−08	–	–	4.8; 4.3	E1; E1 + E2 + E3
	2 A_34244148	1.23E−07	–	–	1.8	E3
	3B_35970259	1.26E−09	–	–	1.4	E1 + E2 + E3
	3B_45474371	1.40E−06; 4.51E−08	–	–	15.1; 29.6	E1; E1 + E2 + E3
	5B_28252956	2.04E−07	–	–	2.5	E1
	**5B_3150126**	1.51E−15; 1.55E−07; 4.16E−14	–	–	22.1; 35.8; 10.6	E1; E2; E1 + E2 + E3
	6 A_40922437	1.92E−07; 1.50E−06	–	–	5.4; 1.9	E2; E1 + E2 + E3
	**6B_24190829**	–	1.66E−06	1.66E−06	60.6	E3
	**6B_40602961**	1.35E−16	–	–	38.1	E3
	7 A_13129745	1.74E−07	–	–	0.8	E1
	7B_49045901	1.61E−06	–	–	6.7	E3
	8B_12513208	2.389E−07	–	–	1.1	E1 + E2 + E3
STG	6B_10864215	1.58E−07	9.90E−08	7.54E−07	21.3	E3
DrtSc	1 A_41691781	2.71E−10	–	–	6.3	E3
	1 A_47690818	1.47E−06	–	–	20.2	E2
	4 A_2499716	5.34E−07	–	–	19.4	E2
	**5B_35573546**	1.02E−07	–	–	33.8	E3
	7 A_42035020	1.72E−07	–	–	10.1	E1
	9 A_6603556	5.02E−09	–	–	13.5	E1
Fe	2B_35126324	–	1.04E-06	1.04E-06	4.6	E2
	2B_35281485*	2.01E−11; 2.38E−08	1.14E-07	1.14E-07	1.9–32.9	E1; E2
	2B_37326032	–	1.05E-06	1.05E-06	2.6	E2
	3B_49895136	3.61E−07	–	–	2.5	E1
	7B_22082060	4.37E−07	–	–	5.0	E1
	8 A_14715050	5.87E−07	–	–	2.8	E1
	8 A_34625287	1.11E−08	–	–	6.2	E1
	9 A_44001813	5.65E−08	–	–	4.5	E1
Zn	2B_35126324	–	1.19E−06	–	12.0	E2
	2B_35281485*	2.76E−27; 2.50E−08	6.31E−07; 5.65E−07	6.31E−07	8.1–30.6; 2.1–8.9	E1; E2
	2B_35342493	-	7.74E−07	–	0.1	E2
	3 A_38743459	3.30E−07	–	–	2.1	E1
	5 A_62694353	9.73E−08	–	–	1.9	E1
	5 A_65700827	2.50E−07	–	–	2.2	E1
	**7B_14800902**	5.00E−13	–	1.61E-06	7.4–44.5	E2
	8B_30935289	1.60E−06	–	–	4.6	E2
	9 A_42427372	9.97E−07	–	–	1.5	E1
	9B_11179336	2.78E−07	–	–	7.3	E1
*Additional Major QTNs detected using Sub-genome A specific MTAs*						
DTF	**8 A_18375210**	3.55E−06	–	–	55.6	E1
PTH	**4 A_3954933**	–	2.01823E-06	2.01823E-06	33.8	E1 + E2 + E3
TSW	**8 A_22146023**	4.92E-09; 5.00E−09	–	–	62.8; 33.8	E1; E1 + E2 + E3
DrtSc	**8 A_23730370**	1.85E−15	–	–	38.7	E3
Fe	**8 A_14715050**	5.56E−07	–	–	42	E1
*Additional Major QTNs detected using Sub-genome A specific MTAs*						
DTF	9B_20268455*		1.37E−06	1.37E−06	35.9	E1 + E2 + E3
DTM	9B_20268455*		1.67E−06	1.67E−06	34.7	E1 + E2 + E3
TSW	**6B_27369367**	–	2.49E−06	2.49E−06	72.3	E1

*Same QTN is reported for Fe and Zn, and for DTF and DTM. SNPs in bold are considered major QTNs reporting >  = 30% PVE in at least one environment

### Whole genome MTAs

**Fig. 5:**
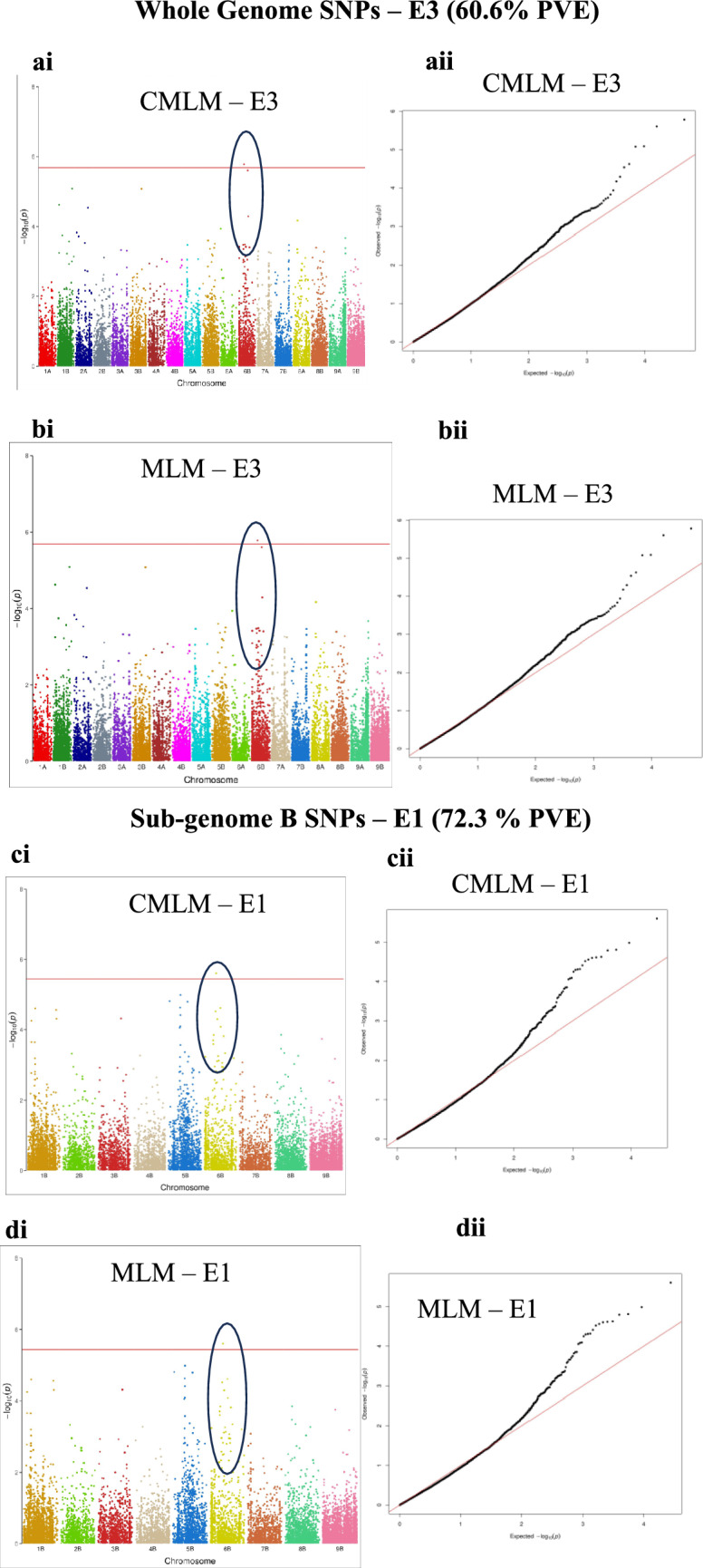
Manhattan and QQ plots showing the TSW major QTNs explaining ~ 60.6% and 72.3% phenotypic variation. **ai & bi.** Manhattan plots drawn using CMLM and MLM models with whole-genome SNPs and showing significant association with TSW for E3. **aii & bii.** The corresponding QQ plot to the Manhattan plot in Ai and Bi plotted based on phenotypic data generated from E3. **ci & di**. Manhattan plots showing the detected major QTN for TSW (72% PVE) for E1 using CMLM and MLM models. **cii & dii**. The corresponding QQ plots to the Manhattan plots in Cii and Dii. Sub-genome A chromosomes are represented by odd numbers, while sub-genome B are represented by even numbers

Fifty-five of the 62 non-redundant QTNs were detected using whole-genome SNPs, of which 8 were major QTNs detected in association with DTM (40.6% phenotypic variation explained (PVE)), TSW (35.8%, 38.1%, 56.1% & 60.6% PVE), DrtSc (33.8% PVE), Zn (44.5% PVE), and Fe and Zn (30.6% PVE; 32.9% PVE) (Table 4). The 4 major QTNs detected for TSW were on different chromosomes; 1B (SNP 1B_64659309; 56.1% PVE), 5B (SNP 5B_3150126; 35.8% PVE), and two on chromosome 6B (SNP 6B_24190829, 60.6% PVE; SNP 6B_40602961, 38.1% PVE) (Fig. 5; Table 4). The highest number of MTAs (13) was reported for TSW, followed by 10 MTAs for Zn and nine for PTH (Table 4). The lowest number was reported for STG, for which the QTN was specifically detected in E3 (Table 4). MTAs were detected in at least one environment for all the traits except STG. One major QTN (SNP 2B_35281485) was detected for both Fe and Zn (Table 4).

### Sub-genome-specific MTAs

Only major QTNs were reported for the sub-genome-specific MTAs in Table 4, while the remaining sub-genome MTAs are reported in Supplementary Table S6. Sub-genome A SNPs detected five additional major QTNs, 3 localized on chromosome 8 A and 1 on chromosome 4 A (Table 4, Supplementary Table S6). The 5 major QTNs were detected for DTF (SNP 8 A_18375210; 55.6% PVE); PTH (SNP 4 A_3954933; 33.8% PVE); TSW (SNP 8 A_22146023; 33.8% and 62.8% PVE), DrtSc (SNP 8 A_23730370; 38.7% PVE) and Fe quality (SNP 8 A_14715050; 42% PVE) (Table 4, Supplementary Table S7). All the major QTNs detected using sub-genome A markers were not detected as major QTNs using whole-genome SNPs (Table 4). Despite having fewer markers than sub-genome B (13,866), the use of sub-genome A markers (10,246) alone added more information and new major QTNs that were not detected while using whole-genome SNPs (Supplementary Table S6).

Sub-genome B SNPs detected additional 2 major QTNs, same SNP (9B_20268455) for DTF (35.9% PVE) and DTM (34.7% PVE), and another SNP (6B_27369367; 72.3% PVE) for TSW (Table 4). The two major QTNs for DTF were only detected when the sub-genome SNPs were used and were totally missed when whole-genome SNPs were deployed for analysis (Table 4). STG MTAs were better detected using sub-genome B or whole-genome SNPs as opposed to sub-genome A SNPs (Supplementary Table S6). The highest PVE by a marker was reported on chromosome 6B (SNP 6B_27369367) for TSW in E1 at 72.3% PVE followed by another marker downstream on the same chromosome (SNP 6B_24190829) for the same trait in E3 at about 60.0% PVE using both CMLM and MLM (Fig. 5).

**Fig. 6:**
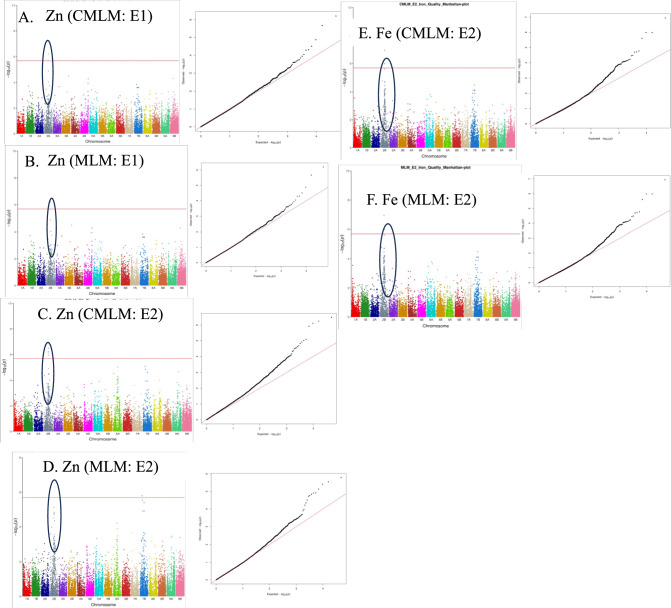
Manhattan and QQ plots revealing the QTN hotspot for Fe and Zn on chromosome 2B. **A–D** Manhattan plots and corresponding QQ plots drawn using Zn quality data from E1 and E2 as shown using CMLM or MLM GWAS models. **E–F**. Manhattan plots and corresponding QQ plots drawn using Fe quality data from E2 as shown using CMLM and MLM GWAS models

A QTN hotspot for Zn and Fe was detected on chromosome 2B using CMLM and MLM (Fig. 6; Table 4). The hotspot QTN was detected across E1 and E2 only (Fig. 6; Table 4) and not in E3.

**Fig. 7:**
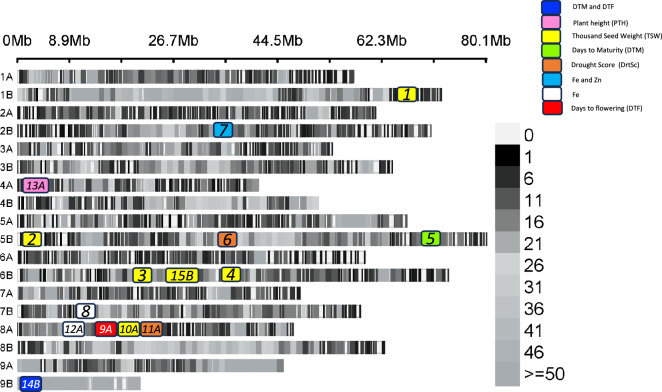
The overall SNP density and the locations of major QTNs for which candidate genes were detected. The SNP density is indicated by the gray to black scale. The color key for each QTN is plotted within the figure. Major QTN locations are estimates and not drawn to scale. Major QTNs detected using sub-genome A SNPs only are denoted with “*A*” and those detected using sub-genome B SNPs only denoted with “*B*”

A major QTN on chromosome 9B (SNP 9B_20268455) for DTF (35.9% PVE) and DTM (34.7% PVE) was detected across the three environments using sub-genome B specific markers (Table 4). All major QTNs were plotted on the finger millet marker density map as shown on Fig. 7. Supplementary Table S6 provides more details of all the significant MTAs with corresponding *P* values, minor allele frequencies (MAF), models, QQ, and Manhattan plots.

### Haplotype analysis

Haplotype analysis of all major QTNs revealed 54 haplotype blocks having significant association with the respective traits of interest (Table 5; Supplementary Table S7). The maximum number of haplotypes detected per locus was 5, while the longest haplotype comprised of 16 nucleotides (Table 5). Detailed information on the haplotypes, the locations of the SNPs, and their frequencies are provided in Supplementary Table S7.

**Table 5:** Haplotypes detected from major QTNs with significant association with the traits

Trait	Major QTN	Haplotypes
DrtSc	1. 5B_35573546	1. GG 2. AG 3. GT
	2. 8 A_23730370	1. GCG 2. ACG 3. GCA 4. GTG
DTF	8 A_18375210	1. CCG 2. TCC 3. CAG 4. CCC
DTF & DTM	9B_20268455	1. CGCCAACAGGAAACTT 2. TGTAGCTGTAGTTTCC 3. CACCAACAGGAAACTT
DTM	5B_65558510	1. CT 2. CG 3. TT
Fe	8 A_14715050	1. AGC 2. CGT 3. CGC 4. CAC
Fe & Zn	2B_35281485	1. AG 2. GA 3. GG
Zn	7B_14800902	1. CGG 2. TGA 3. TAA
PTH	4 A_3954933	1. TTCC 2. CCGT 3. TTGT
TSW	1. 1B_64659309	1. GAG 2. AAG 3. GAA 4. GGG
	2. 5B_3150126	1. TGCGG 2. TGTAA 3. GGCGG 4. TACGG
	3. 6B_24190829	1. GAGGT 2. GGGAG 3. GGGGG 4. AAGGT 5. GGAGG
	4. 6B_27369367	1. GGC 2. AAC 3. GGG
	5. 6B_40602961	1. ACGCG 2. GCGTG 3. GTGCG 4. ACACG 5. GCGCG
	6. 8 A_22146023	1. AGCC 2. AGCT 3. GATC

**Fig. 8:**
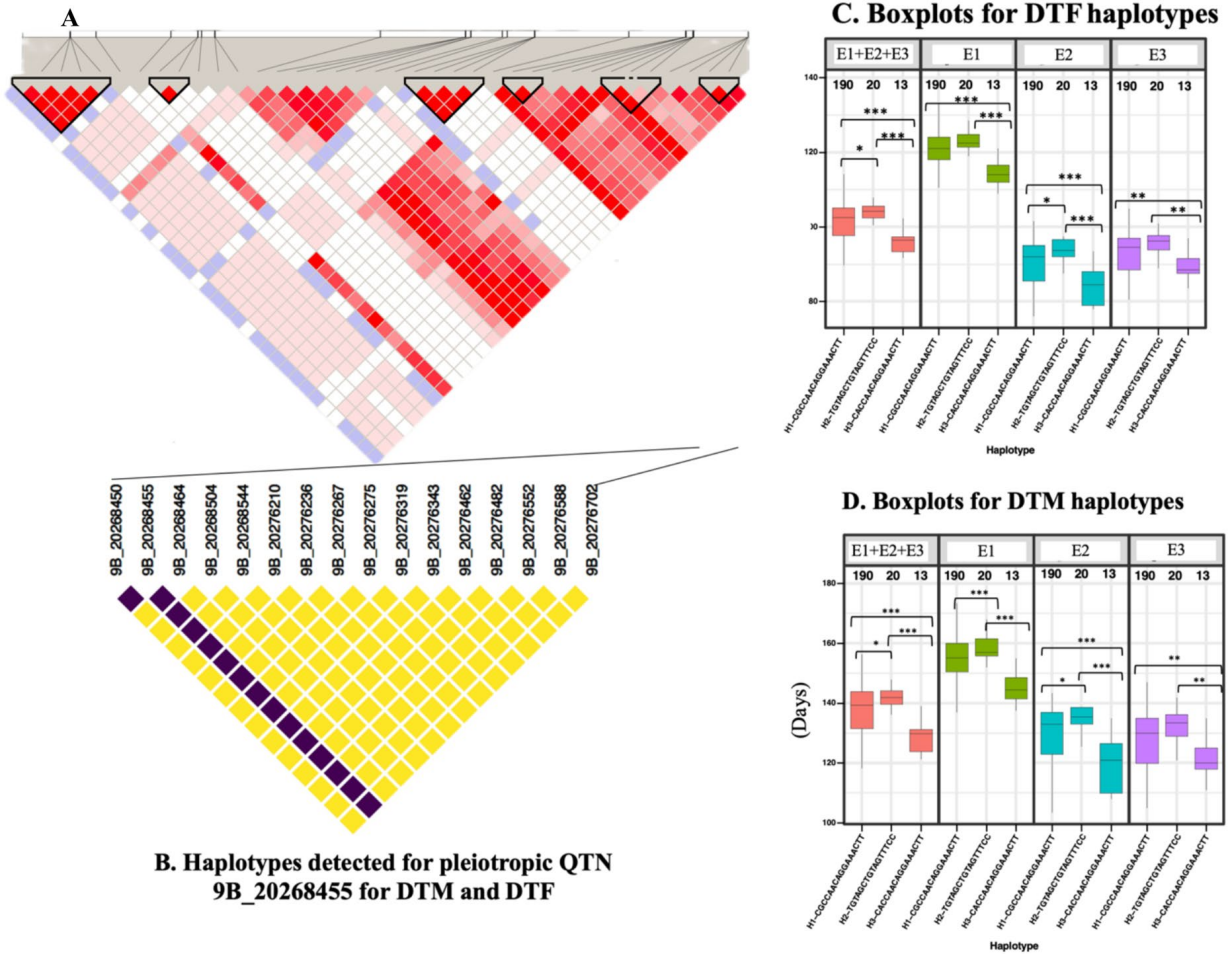
Graphical representation of haplotype blocks across chromosome 9 and around the pleiotropic QTN 9B_20268455 for DTM and DTF. **A.** Haplotype blocks across the whole chromosome 9B. **B.** Haplotypes detected for pleiotropic QTN 9B_20268455 for DTM and DTF.** C.** Boxplots showing the performance of genotypes harboring various haplotypes with respect to DTF. **D.** Boxplots showing the performance of individual genotypes harboring various haplotypes with respect to DTF. All haplotypes are significantly different at *P < 0.05; **P < 0.01; ***P < 0.001 (Supplementary Table S8)

One way ANOVA of different haplotype pairs detected 195 significant associations with target traits, majority (136) of which were significant at *P* < 0.001, 35 at *P* < 0.05, and 24 at *P* < 0.01 (Supplementary Table S8). Box plots showing comparisons between pairs of haplotypes at each test location and across environments revealed that most of the favorable haplotypes were rare and present in a smaller percentage of individuals (Supplementary Fig. S4). Pleiotropic haplotypes associated with both DTM and DTF identified on chromosome 9B revealed the largest block containing 16 SNPs (Fig. 8). The most favorable haplotype for DTM and DTF at the pleiotropic QTN on chromosome 9B (9B_20268455) was H3 (CACCAACAGGAAACTT), which was present in 13 individuals (Fig. 8C and D).

**Fig. 9:**
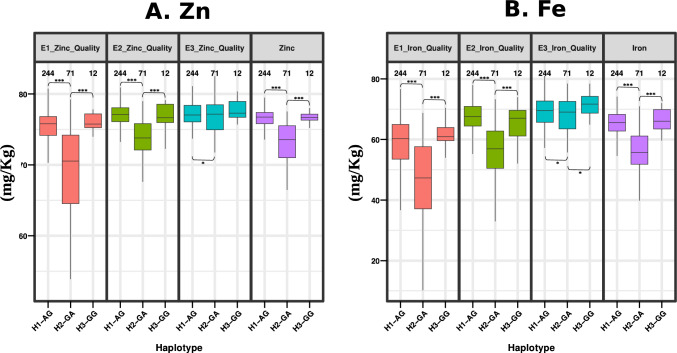
Boxplots showing differences in performances of individuals harboring different haplotype blocks generated based on a major QTN that was associated with both Fe and Zn. **A.** Differences in haplotypes detected for pleiotropic QTN 2B_35281485 for Zn. **B.** Differences in haplotypes detected for pleiotropic QTN 2B_35281485 for Fe. All haplotypes are significantly different (Supplementary Table S8)

The other pleiotropic haplotype associated with Fe and Zn was revealed on chromosome 2B (2B_35281485), was having two nucleotides, with the most favorable haplotype being GG that was present in 12 individuals (Fig. 9).

### Regional distribution of genotypes having favorable haplotypes around the pleiotropic QTNs

Using data across the 3 environments, majority of the 13 and 12 genotypes harboring favorable haplotypes for DTM/DTF and Fe/Zn respectively were from Tigray region (Table 6), consistent with the enrichment of superior performing genotypes in the same region (Table 2 and 3). Three genotypes (243635, AxumARCColl-25 and AxumARCColl-31) uniquely harbored both the DTM/DTF and Fe/Zn favorable haplotypes (Table 6). Only one released variety (Tekeze-1) was among the list of genotypes having superior pleiotropic haplotypes (Table 6).

**Table 6:** Distribution of genotypes harboring favorable pleiotropic haplotypes for DTM/DTF and Fe/Zn

Trait & locus	Haplotype	Genotypes	Region of collection
DTM/DTF 9B_20268455	H3: CACCAACAGGAAACTT	100065	Oromiya
		208442	Amara
		211029	Tigray
		215850	Amara
		215887	Amara
		234186	Tigray
		237473	Tigray
		243635*	Amara
		AxumARCColl-25*	Tigray
		AxumARCColl-27	Tigray
		AxumARCColl-31*	Tigray
		Tekeze-1	Tigray
Fe & Zn 2B_35281485	H3: GG	219831	Tigray
		234185	Tigray
		235153	Tigray
		238337	Tigray
		243635*	Amara
		AxumARCColl-13	Tigray
		AxumARCColl-25*	Tigray
		AxumARCColl-28	Tigray
		AxumARCColl-29	Tigray
		AxumARCColl-31*	Tigray
		AxumARCColl-35	Tigray
		AxumARCColl-3	Tigray
		AxumARCColl-7	Tigray

*Genotypes having both DTM/DTF and Zn/Fe favorable haplotypes

### Functional QTNs and gene-specific haplotypes

**Fig. 10:**
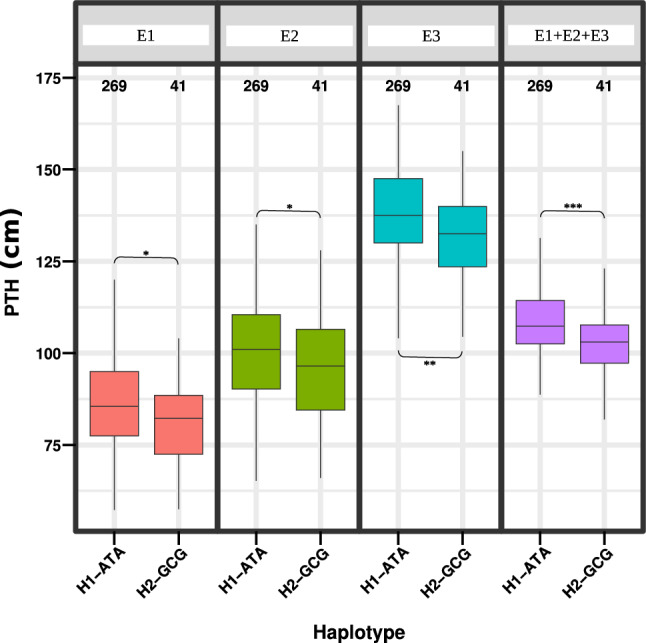
Boxplots showing differences in performances of individuals harboring the two different haplotypes for PTH within the gene ELECO.r07.4 AG0303600 associated with plant height

Ten genes were found to co-localize with major QTNs, two of which contained more than 1 SNP (Chromosomes 4 A and 9B; Table 7). Four of the genes were associated with PTH, one gene (*glycosyltransferase*) with TSW, two genes with Fe and one gene with DTM/DTF (Table 7). The two genes that harbored at least two SNPs were *ABC transporter family protein* (ELECO.r07.4 AG0303600) and *ankyrin repeat family protein* (ELECO.r07.9BG0703480) and were associated with PTH and TSW (Table 7). The study identified 2 haplotypes (H1-ATA, H2-GCG) for ELECO.r07.4 AG0303600 and 4 haplotypes (H1-CCGGCGAG, H2-TTAACAGA, H3- CCGGTGAG, H4-CTAACAGA) for ELECO.r07.9BG0703480 (Supplementary Table S9). Mean comparison tests between haplotypes of ELECO.r07.9BG0703480 revealed no significant differences (*P* < 0.05) between genotypes (Supplementary Table S10) and were therefore dropped from further analysis. Significant association between ELECO.r07.4 AG0303600 haplotypes were observed in E1 (*P* < 0.05), E2 (*P* < 0.05), E3 (*P* < 0.01) and across environments (*P* < 0.001) (Supplementary Table S9; Fig. 10).

**Table 7:** Genes colocalizing with major QTNs

Gene Id	Gene description	Trait	Allele	Pos
ELECO.r07.4 AG0303570	RPM1-interacting protein 4-like protein	PTH	C/G	3,404,460
ELECO.r07.4 AG0304200	Polygalacturonase QRT3	PTH	C/T	3,940,479
ELECO.r07.4 AG0304230	30S ribosomal protein S17	PTH	C/T	3,955,182
ELECO.r07.4 AG0304420	Shikimate/quinate hydroxycinnamoyl transferase	PTH	T/C	4,204,724
ELECO.r07.4 AG0303600*	Multidrug resistance protein ABC transporter family protein	PTH	A/G	3,454,764
			T/C	3,454,863
			A/G	3,454,904
ELECO.r07.5BG0413640	Glycosyltransferase	TSW	G/A	2,841,435
ELECO.r07.8 AG0623600	4'-phosphopantetheinyl transferase	Fe	C/T	14,459,510
ELECO.r07.8 AG0623750	Receptor kinase 1	Fe	T/A	15,284,945
ELECO.r07.9BG0703480*	Ankyrin repeat family protein	DTF and DTM	C/T	19,836,545
			C/T	19,836,552
			G/A	19,836,556
			G/A	19,836,782
			C/T	19,836,804
			G/A	19,836,808
			A/G	19,836,813
			G/A	19,836,895
			G/A	20,349,730

*Highlights genes for which gene-based haplotype analysis was done

### Identification of all other candidate genes from major QTNs using whole-genome and sub-genome A SNPs

All other candidate genes identified within major QTNs were retrieved and assembled in Supplementary Table S10. Three hundred and nineteen (319) genes were retrieved from chromosomes 1B (TSW), 2B (Fe and Zn), 5B (DrtSc and DTM), 6B (TSW), and 7B (Zn content) based on whole-genome SNP analysis, and a further one hundred and forty nine (149) candidate genes (15 for DTF; 4 for TSW; 8 for DrtSc, 23 for Fe and 99 for PTH) from using sub-genome A SNPs only and 10 genes (6 for DTF and DTM; 4 for TSW) from using sub-genome B SNPs only (Supplementary Table S10). The four major QTNs for TSW detected using whole-genome SNPs revealed 269 candidate genes, with the first QTN on chromosome 1B having a cluster of *Late embryogenesis abundant (LEA) hydroxyproline-rich glycoprotein* family and *Katanin p80 WD40 repeat-containing subunit B1 homolog* (Supplementary Table S10)*.* The second TSW major QTN on chromosome 5B reported an abundance of *Ubiquitin carboxyl-terminal hydrolase* 12, *O-methyltransferase-like protein* and *glycosyltransferases,* while the third TSW QTN revealed abundance of *serpins* (Supplementary Table S10). An additional *Ubiquitin family protein* was also identified in the TSW major QTN on chromosome 8 A that was detected using the sub-genome A SNPs only (Supplementary Table S10). The additional candidate genes detected for TSW on chromosome 6B contained four genes, two of which had been detected in other TSW QTNs. GDSL esterase/lipase gene detected on chromosome 6B for TSW was also detected on chromosome 5B major QTN for TSW; GRF zinc finger family protein was also detected upstream within LD of another TSW QTN (6B_40602961).

The DTM QTN showed abundance of *Calmodulin-binding protein,* as well as *Transmembrane protein* families. Two additional *Transmembrane protein* families were identified for DTF on chromosome 8 A using sub-genome A SNPs. The major QTN for Fe detected using sub-genome A markers revealed a cluster of 3 *Cysteine protease genes* that were ~ 0.5 Mb upstream of the significant marker (Supplementary Table S10). The same Fe major QTN on chromosome 8 A also harbored 2 *cytochrome P450* genes and one *Cytochrome P450 reductase* (Supplementary Table S10). One *Receptor protein kinase* was reported for Fe major QTN on chromosome 7B, while 2 *Receptor protein kinases* were reported for the same trait on chromosome 8 A (Supplementary Table S10). All enriched candidate genes have been highlighted in yellow in Supplementary Table S10 for ease of detection.

## Discussion

The findings of this research demonstrate tremendous genetic variation for both agronomic and nutrition-related traits for Ethiopian finger millet germplasms evaluated across three different environments. Our study is the first comprehensive GWAS for both agronomic and nutrition-related traits for finger millet in Africa using more than 300 genotypes. Beyond identifying significant MTAs, we also identified promising candidate genes and favorable haplotypes for the target traits that could be deployed right away into breeding programs. Overall, results confirm earlier studies that there is adequate genetic variation among finger millet germplasm to select for several traits such as drought, agro-morphological, and micronutrient content (Lule et al. 2012; Assefa et al. 2013; Backiyalakshmi et al. 2023; Chandra et al. 2024).

### Phenotypic variation for the target traits

The present investigation revealed highly significant phenotypic variation between genotypes for all the traits studied across and within each of the locations. The current findings confirm that the assembled set of genotypes were ideal for trait discovery and paves the way for their utilization in other studies. Ethiopia is considered a primary center of diversity for finger millet (Purseglove 1985; Tsehaye et al. 2006; Tesfaye and Mengistu 2017), therefore likely to harbor important agronomic diversity required for crop improvement. Confirming this high extent of agronomic diversity among Ethiopian germplasm was also important for finger millet, in which the yields are extremely low and the gap between potential yield (6–8 tons/ha) and farmers’ yields (< 2.5 tons/ha) is extremely high (Gebreyohannes et al. 2021, 2024). Our study identified unimproved material yielding much more than the released cultivars, especially in Arsinegelle, suggesting that the existing native agronomic potential of Ethiopian finger millet has not been exploited to the farmers’ advantages. Furthermore, most of the top ten best yielders and those harboring the highest concentration of micronutrient content were from Amhara and Tigray, the two regions from which the study could confirm minimal clustering with released varieties. Therefore, future efforts will need to introduce these promising landraces into breeding programs and design relevant agronomic and management packages that are appropriate for farmers in enhancing their yields (Beres et al. 2020).

Relatively high broad sense heritability (*H*^*2*^*)* for most of the traits was identified in each of the environments, an indication of greater genetic control for the traits studied. Previous studies in finger millet that included at least 100 genotypes also reported similar *H*^*2*^ values for some of the traits such as DTF (Lule et al. 2012; Eric et al. 2016), DTM (Anteneh et al. 2019), GY (Owere et al. 2015; Eric et al. 2016), PTH (Lule et al. 2012; Eric et al. 2016), and TSW (Lule et al. 2012). To the best of our knowledge, this was the first reported large-scale screening for the stay-green trait (STG) in finger millet, for which very high *H*^*2*^ values were recorded. The stay-green trait has been used in several crops, including sorghum (Borell et al. 2014), to select for drought tolerance but is yet to be exploited in finger millet. Recording high *H*^*2*^ values is promising and indicates the potential for genetic improvement of drought tolerance in finger millet using STG. Significant correlation between STG and drought score was also observed in E2 and E3, suggesting that the observed STG contributes to drought tolerance in these sites. More studies will need to be done to ensure that the stay-green trait being introduced into breeding programs is functional (Jordan et al. 2012) and not cosmetic (Myers et al. 2018).

The NIR-based estimates of Fe and Zn showed a moderate level of *H*^*2*^ (0.5–0.7). This is a good level of accuracy considering the prohibitive nature of the costs of wet chemistry. Although these results will still need to be validated through wet chemistry, the range of Fe content recorded in the current study was higher than reported for the global finger millet core collection (Upadhyaya et al. 2010) but comparable to Puranik et al. (2020), who analyzed a diverse set of finger millet genotypes that had been evaluated in Kenya. The Zn content reported in this investigation exceeds most of the previous reports and only comparable to previous studies in which plants had been grown in Zn treated soils (Yamunarani et al. 2016; Teklu et al. 2024). A recent study on agronomic biofortification of Fe and Zn in Ethiopia reported up to 21.4% increase in grain Fe and Zn content following the application of Zn and Fe fertilizer (Teklu et al. 2023b). Previous studies reported higher Fe grain concentrations than Zn (Upadhyaya et al. 2010; Puranik et al. 2020; Fred et al. 2021; Teklu et al. 2023b) in contrast to our study where Zn concentrations were higher. The relatively higher Zn concentrations were consistent across the 3 locations and therefore reveal a likely unique germplasm set for improving grain Zn content in breeding programs. Our results might also suggest that there could be abundant genetic variation for Zn content in Ethiopia that may not have been captured within the global collections.

### Genetic variation, population structure, and linkage disequilibrium

We stringently filtered our markers to retain a highly informative marker set for further characterization of the genotypes. The study also chose to conduct all the genetic analysis using whole genome, sub-genome A and sub-genome B sets of markers, following interesting results from previous studies (Devos et al. 2023; Bančič et al. 2023). The retained markers explained more than 45% genetic variation between the genotypes and successfully defined four sub-populations across the germplasm irrespective of which marker set was used. The marker density of 16 SNPs/Mb was twice higher than reported in Bančič et al. (2023). Retaining an informative set of SNP markers is a pre-requisite for population structure analysis and for determining differences between individuals. The results of the present study successfully showed that most of the genotypes from Tigray and Amhara regions have not been actively used in breeding programs based on the minimal clustering of the released varieties within the Tigray and Amhara clusters. The germplasm used is, therefore, a unique resource that should be introduced into breeding programs to enrich the pool of breeding lines, and will also need to be well maintained and conserved to ensure the allelic richness is available in the future. Being native to Ethiopia, there is likely to be diverse cultivated and wild finger millet accessions that are growing alongside each other with regular gene flow between them (Tsehaye et al. 2006; Tesfaye and Mengistu 2017; Lule et al. 2018; Dida et al. 2021; Pendergast et al. 2022). Gene flow can enhance adaptation and increase genetic variation for traits of interest that should be regularly captured through focused collection missions. Despite being an important center of diversity for finger millet, there are currently only ~ 30 finger millet genotypes from Ethiopia that are conserved in the ICRISAT global germplasm collection (ICRISAT 2024). This study reveals a need to have better representation of both Tigray and Amhara regions finger millet collection and characterization within the local and global gene banks.

All the three sets of markers revealed four sub-populations using DAPC analysis, although the sub-genome marker sets revealed more distinct clusters. The four clusters are consistent with Bančič et al. (2023) who studied 423 cultivated finger millet accessions from India, Nepal, East Africa, and Southern Africa using whole-genome SNPs. Devos et al. (2023) performed a similar analysis with the addition of wild accessions and reported two distinct clusters irrespective of which marker set was used. A repeat analysis of the same dataset in addition to existing datasets of wild accessions will be interesting in confirming the results reported by Devos et al. (2023). The clear difference in our study was the ability of the sub-genome markers to separate the sub-populations more clearly than the whole-genome SNPs.

We also observed a huge difference in the LD decay points between the two sub-genomes, which would need to be studied further in more diverse germplasm sets. Using naïve models, the study confirmed that LD decayed more rapidly in the sub-genome A (1.2 Mb) in comparison to sub-genome B (3.2 Mb), which is consistent with a previous study in which LD decayed much faster in the sub-genome A with or without genetic structure correction (Bančič et al. 2023). LD is important in association mapping as it determines the level of optimal resolution required for an association study. Although LD has not been studied much in finger millet, the reported slow decay is not surprising given the highly self-pollinating nature of this crop. The difference in LD between the two sub-genomes could also be traced back to the progenitors. It is known that the A sub-genome donor is a wild weedy species, *Eleusine indica* (Zhang et al. 2019), hence a more rapid decay would be expected. LD decays more rapidly in wild populations than in cultivated crops (Sela et al. 2014).

### Marker-trait associations, haplotype analysis, and candidate genes

Our stringent filtering of markers and the use of Bonferroni threshold paid off in detecting major QTN regions. Some of the major QTNs detected in the current study were extremely novel and will form the basis for routine breeding and further research on the respective traits. Bančič et al. (2023) and Sood et al. (2023) detected several MTAs while studying agronomic traits in finger millet, but none of the reported associations explained > 20% phenotypic variation. Our study revealed major QTNs for DTM, DTF, TSW, DrtSc, Fe, and Zn alongside several other significant QTNs. The major QTNs were associated with economically important traits in finger millet, all of which are still being improved using traditional methods for most part. We additionally chose a stringent 30% PVE as a cut-off for major QTNs to enhance the immediate application of our results into molecular breeding. There are studies reporting as low as 10% PVE as major QTLs (Pandey et al. 2020). A major QTL/QTN is one that is detected in more than one environment and has a large effect on the phenotype. Most studies recommended at least 15 to 20% PVE for the locus to be considered as major (Du et al. 2015). This study employed a high threshold of 30% PVE as a cut-off to reduce the numbers of false positives when the associated markers are introduced into breeding programs for genomics-assisted selection and introgression. While recent advances in next-generation sequencing (NGS) have led to the development of quality control (QC) markers in finger millet, markers associated with economically important traits are still lacking. These major QTNs will add significant value toward early generation and genomic selection in structured finger millet breeding programs.

The potential reliability of the major QTNs for breeding was further validated using haplotype analysis. Haplotype analysis has been reported to improve the identification of genes of interest in other cereal crops including rice (Yang et al. 2023) and wheat (Taranto et al. 2023) but not yet exploited in finger millet, partly due to the previous lack of high-quality reference genome. Using a chromosome scale reference genome (Devos et al. 2023), we detected significant associations of different haplotype pairs with the target traits across all major QTNs. Associated haplotypes would enhance the precision of selection and introgression and improve breeding efficiency. Our results also provide opportunity to stack different favorable haplotypes into superior elite backgrounds for better performance. In some cases, we identified individuals harboring more than one favorable haplotypes making such genotypes obvious candidates for immediate release, or introduction into various breeding programs. While a more comprehensive haplotype analysis of all QTNs identified will still be required to demonstrate the potential benefits of the QTNs detected, the results obtained from major QTLs form a baseline from which future similar studies will be anchored. Haplotype based breeding (HBB) promises to enhance genetic gain (Sivabharathi et al. 2024), especially in an under-researched crop such as finger millet.

The highest numbers of major QTNs were reported for TSW, which is one of the important determinants of grain yield in finger millet (Wolie and Dessalegn 2011). Haplotype analysis for seven major QTNs for TSW revealed clear differences in haplotypes demonstrating the readiness of all the markers identified for immediate use in breeding programs. Low yields in finger millet have been reported as one of the limiting factors to commercialization of the crop. Field evaluation, genetic diversity, and haplotype analysis results provide new knowledge and reveal that grain yield in finger millet has not been studied and exploited sufficiently. In rice (*Oryza sativa* L.), where seed weight has been extensively studied, three major genes have been reported for TSW. The first TSW locus in rice, *GS3*, encodes a transmembrane protein (Fan et al. 2006), while the second, *GW5*, encodes a calmodulin binding protein (Liu et al. 2017). A third locus encodes a transcription repressor *OsBZR1*, which represses *FZP* (*Frizzy Panicle*) expression (Bai et al. 2017). This study identified 3 copies of *calmodulin binding proteins* and 4 *transmembrane proteins* in the DTM major QTN. DTM was found to be positively correlated with TSW. A major TSW QTN was also found colocalized with a *glycosyltransferase* gene on chromosome 5B in our study. These interesting candidate genes make a priority list for functional validation toward increasing grain size and yield in finger millet. Finger millet researchers will now have the favorable haplotypes and unique high yielding landraces to deploy in their programs and improve genetic gain as soon as research investments are made available.

Gene-specific haplotype analysis done for two major QTNs reported promising results and suggests that future studies should consider all the significant QTNs for gene-specific haplotype analysis in the future. The two haplotypes identified for *ELECO.r07.4 AG0303600* for PTH are promising for immediate deployment in selection for and/or against the trait. Tall plants in finger millet have been associated with lodging, which is one of the breeding objectives (Lule et al. 2012). Wolie and Dessalegn (2011) reported negative correlation between PTH and grain yield (dwarf plants are better yielding) and a positive correlation with biomass. It has been argued that the introduction of dwarf plants into cereals may have saved more lives than any other scientific development (Hedden 2003). The clear differences in PTH of each of the haplotype pools is proof that this gene-specific haplotype has the potential to play a huge role if introduced into breeding programs, either for grain yield or biomass. Haplotype GCG of *ELECO.r07.4 AG0303600* should be studied further alongside TSW haplotypes for improving yields. Finger millet for fodder also remains an underexploited field that could take advantage of haplotype ATA of the gene *ELECO.r07.4 AG0303600. ELECO.r07.4 AG0303600* codes for ABC transporter gene family that is not well characterized in finger millet. However, there are several reports in many crops demonstrating the involvement of ABC transporter genes in dwarfism (Busov et al. 2008; Wang et al. 2017; Ye et al. 2013; Zhu et al. 2019). The identification of a putative ABC transporter in PTH opens a new chapter for both haplotype-based breeding and functional characterization to confirm its role in finger millet.

Drought tolerance is also an important trait in finger millet that can result in significant yield penalty (Krishnamurthy et al. 2016). This study identified a major QTN for drought tolerance and a relatively minor one for the stay-green trait (21.3% PVE). Haplotype analysis on the major QTNs for DrtSc on chromosomes 5B and 8 A revealed highly significant haplotypes that should be deployed right away in breeding programs. The most applicable would be the negative selection against drought sensitive haplotypes, which were very consistent across environments. The stay-green QTN will also need to be explored further because it revealed significant contribution to the phenotype. Natural variation in the promoter region of a rice stay-green gene has been exploited in breeding and led to delayed senescence and increased grain yield (Shin et al. 2020). Similar studies can be designed in finger millet to further characterize the stay-green QTN alongside the stable DrtSc QTNs and haplotypes.

The pleiotropic QTNs detected between DTM and DTF and between Fe and Zn were not surprising. There is evidence of positive correlation between Fe and Zn in pearl millet (Govindaraj et al. 2022), finger millet (Ojulong et al. 2021), as well as in several other crops (Cichy et al. 2009; Velu et al. 2017; Singh et al. 2018; Sanjana Reddy et al. 2010; Diaz et al. 2022). Our results further confirm a common major QTN for both traits in finger millet for the first time providing more evidence for joint genomics-assisted selection for Fe and Zn. Malnutrition has been identified among the top priority problems that result in high levels of maternal and infant mortality in Ethiopia (Kassebaum et al. 2014). The QTNs identified here can be immediately deployed for the selection of genotypes enriched for the two elements. Once validated, such markers could also be used in the varietal release process as a standard to ensure only high Fe and Zn varieties are released, or recommended as a biofortification solution to the malnutrition cases, not only in Ethiopia, but in all finger millet producing countries. Both DTF and DTM are traits responsible for earliness, therefore a confirmation of a common QTN is a great validation of our results. Immediate validation and introduction of these markers will make it possible to improve the correlated traits simultaneously.

All the major QTNs detected while using whole-genome SNPs were from the sub-genome B. The major QTNs on chromosome 8 A were only detected when sub-genome A markers alone were used in the QTN analysis. Bančič et al. (2023) also detected significant QTNs only in the sub-genome B when whole-genome SNPs were used in the analysis. This result was not surprising given the differences observed in LD pattern for the different sub-genomes, as well as for the whole-genome SNPs. The appropriate MTA threshold would vary depending on both population- and genome-specific factors such as LD pattern and minor allele frequency (MAF) (Kaler and Purcell 2019). Given the rapid rate of LD decay in sub-genome A and the relatively fewer numbers of markers, the Bonferroni threshold used for the whole-genome SNPs may have resulted in several false negatives for sub-genome A that could be detected when sub-genome A SNPs alone were used for the analysis. Although there are not many GWAS reports for finger millet using cultivated species that distinguish the two sub-genomes, it will be important for future studies to take this point into consideration in order to ensure that no potential associations are missed out.

## Conclusion

This study significantly advanced our understanding on the genetic basis of important agronomic and nutrient traits in finger millet. The use of skim-sequencing SNPs alongside a comprehensive and stringent GWAS approach identified major QTNs, favorable haplotypes, and promising candidate genes which are all novel discoveries in finger millet. To ensure the identified MTAs, haplotypes and candidate genes translate into practical applications, allelic and functional characterization will be required for their validation. Once validated, the markers reported in the current study will be great candidates for genomics-assisted selection and haplotype-based breeding in finger millet, in which there are currently none. To enhance conservation efforts and promote utilization in global breeding programs, further characterization of the Ethiopian finger millet germplasm is recommended. Ethiopian breeding program will likely benefit tremendously from integrating exotic germplasm into their conservation and breeding programs. Simultaneous improvement of agronomic and nutritional traits in finger millet breeding programs will pave the way toward making finger millet the desired global food security and climate resilient crop.

## Supplementary Information

Below is the link to the electronic supplementary material.

Supplementary file 1 (XLSX 19450 KB)

Supplementary file 2 (DOCX 2721 KB)

## Declarations

### Data availability

The datasets generated and/or analyzed during the current study are available from the corresponding author on reasonable request.
