# Supplementary material for: Genetic structure of Ethiopian finger millet landraces and genome-wide association mapping for agronomic and nutritional traits
Source: Theor Appl Genet. 2025 May 8;138(6):111. doi: 10.1007/s00122-025-04892-1 (PMC12062187; doi:10.1007/s00122-025-04892-1)
Supplement: Supplementary file 2 — (DOCX 2721 KB) [file 122_2025_4892_MOESM2_ESM.docx]

**Supplementary Fig. S1** A map of Ethiopia showing finger millet landraces collection regions, including Tigray, Amhara, Benishangul Gumz, Oromia and Southern Nation, Nationalities, and People (SNNP). The number of genotypes from each region is indicated in brackets. The evaluation sites are designated as E1= Arsinegelle, E2= Maitsebri, and E3= Meiso.

**Supplementary Fig. S2** A picture showing the staygreen phenotype in finger millet. The mature fingers can be seen on plants with majority green leaves.

**Supplementary Fig. S3** LD decay plots showing differences in the LD decay distance. **a.** Whole genome overall LD decay distance of 2.1 Mbp. **b.** LD decay distance of 1.2 Mb for sub-genome A. **c.** LD decay distance of ~3.2 Mb for sub-genome B.


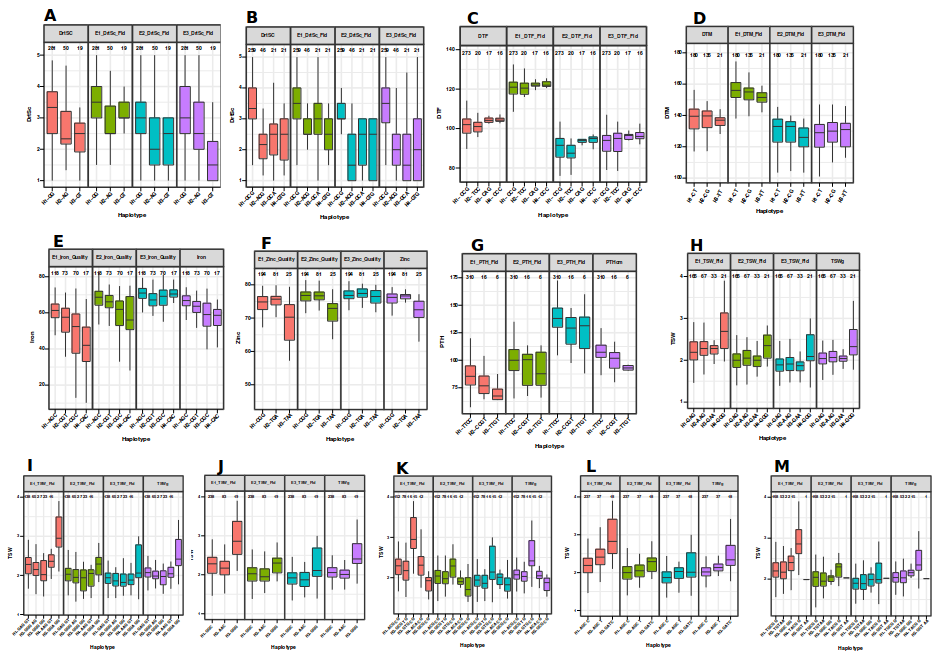


**Fig S4** Boxplots showing differences in performances of individuals harboring different haplotype blocks generated from major QTNs. **A**. DrtSc haplotypes detected for chromosome 5B (5B_35573546) **B.** DrtSc haplotypes detected for chromosome 8A (8A_23730370) **C**. DTF haplotypes detected for chromosome 8A (8A_18375210). **D.** DTM haplotypes detected for chromosome 5B (5B_65558510). **E.** Fe content haplotypes detected for chromosome 8A (8A_14715050). **F.** Zn content haplotypes detected for chromosome 7B (7B_14800902). **G.** PTH haplotypes detected for chromosome 4A (4A_3954933). All TSW haplotypes detected were plotted for 1B_64659309 (**H**), 5B_3150126 (**I**), 6B_24190829 (**J**), 6B_27369367 (**K**), 6B_40602961 (**L**) and 8A_22146023 (**M**).
